# Analysis of Complexome Profiles with the Gaussian Interaction Profiler (GIP) Reveals Novel Protein Complexes in *Plasmodium falciparum*

**DOI:** 10.1021/acs.jproteome.4c00414

**Published:** 2024-09-12

**Authors:** Joeri van Strien, Felix Evers, Alfredo Cabrera-Orefice, Iris Delhez, Taco W. A. Kooij, Martijn A. Huynen

**Affiliations:** † Department of Medical BioSciences, Radboud University Medical Center, 6500 HB Nijmegen, The Netherlands; ‡ Medical Microbiology, Radboud Community for Infectious Diseases, Radboud University Medical Center, 6500 HB Nijmegen, The Netherlands

**Keywords:** complexome profiling, clustering, protein complex, proteomics, *Plasmodium*

## Abstract

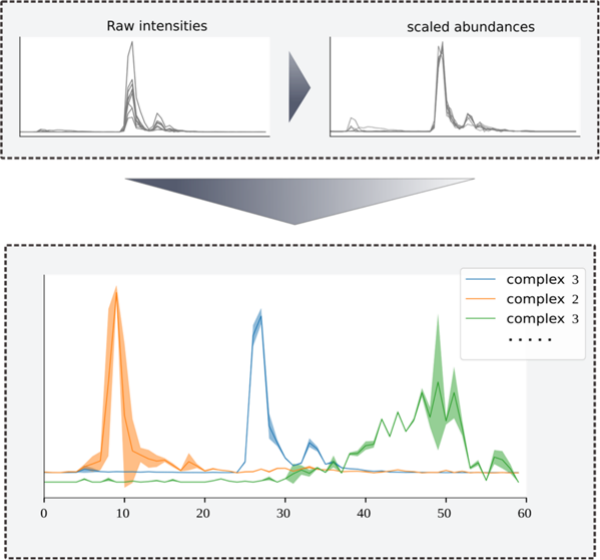

Complexome profiling is an experimental approach to identify interactions by integrating native separation of protein complexes and quantitative mass spectrometry. In a typical complexome profile, thousands of proteins are detected across typically ≤100 fractions. This relatively low resolution leads to similar abundance profiles between proteins that are not necessarily interaction partners. To address this challenge, we introduce the Gaussian Interaction Profiler (GIP), a Gaussian mixture modeling-based clustering workflow that assigns protein clusters by modeling the migration profile of each cluster. Uniquely, the GIP offers a way to prioritize actual interactors over spuriously comigrating proteins. Using previously analyzed human fibroblast complexome profiles, we show good performance of the GIP compared to other state-of-the-art tools. We further demonstrate GIP utility by applying it to complexome profiles from the transmissible lifecycle stage of malaria parasites. We unveil promising novel associations for future experimental verification, including an interaction between the vaccine target Pfs47 and the hypothetical protein PF3D7_0417000. Taken together, the GIP provides methodological advances that facilitate more accurate and automated detection of protein complexes, setting the stage for more varied and nuanced analyses in the field of complexome profiling. The complexome profiling data have been deposited to the ProteomeXchange Consortium with the dataset identifier PXD050751.

## Introduction

Complexome profiling is an experimental approach that, through biochemical separation of proteins in their native state and further fractionation, allows investigation of the presence and composition of protein complexes.^1−3^ The resulting fractions are analyzed using mass spectrometry-based bottom-up proteomics, determining the presence and abundance of proteins in each separated fraction. The protein signals in the separate fractions are then combined to reconstruct the abundance pattern of each detected protein, henceforth referred to as their migration pattern. These are then analyzed using various computational approaches to study the presence, composition, and assembly of complexes. The computational methods used to identify and characterize protein complexes from these data employ various clustering-based approaches. They can generally be divided into two classes.

The first class clusters the detected proteins based on their complete migration patterns.^4−8^ Various metrics can be computed to assign values to the similarity between two migration patterns. Commonly used features include, among others, correlation metrics between the complete migration patterns and metrics based on shared peaks in the migration patterns. In some cases a single metric is computed and used as input for clustering,^4,5^ while, in others, machine learning algorithms are used to integrate various metrics to generate an improved interaction score prior to clustering.^6−8^ These machine learning approaches can also be used to integrate external evidence of interaction in addition to metrics derived from complexome profiling.^9−11^ However, a specific protein complex is usually represented as a single peak at a specific location in the migration patterns of its constituent proteins rather than a full migration pattern shared between the constituent proteins. As this class of approaches does not utilize the fact that a specific subset of fractions represents a complex, comigration of other areas of the migration patterns, irrelevant to this specific interaction can result in incorrect or missed interaction predictions.

The second class identifies individual peaks present in each protein migration pattern using various peak deconvolution approaches,^12−14^ addressing the aforementioned issue. These individual peaks are then clustered to identify possible protein complexes, rather than clustering detected proteins based on the entire migration pattern. However, proteins that form a protein complex often share more than one peak in the migration pattern, due to the presence of subcomplexes, assembly intermediates of the complex, as well as superassemblies, consisting of a multimer of the complex or a supercomplex of the given complex attached to other complexes. This information could help distinguish true interactors from spuriously comigrating proteins but is disregarded when clustering individual peaks.

In addition to the aforementioned limitations of existing analysis approaches, a typical complexome profile contains a large number of proteins (often multiple thousands) that are resolved across a limited number of typically around 60 fractions, causing many proteins that are not interacting to be abundant in the same fractions. After cluster analysis, this in practice results in a large number of clusters of which many do not represent actual protein complexes, while those that do represent complexes can contain spuriously comigrating proteins that do not interact alongside actual complex subunits. While existing approaches have proved useful in characterizing known or conserved complexes from complexome profiling data, identifying novel putative complexes and unambiguously determining the composition of complexes remains a challenge due to the lack of means to prioritize true interactors and complexes.

Therefore, we introduce the Gaussian Interaction Profiler (GIP): a Gaussian mixture modeling-based workflow to identify complexes from a complexome profiling data set. Using Gaussian mixture modeling, the algorithm models the migration profile of putative protein complexes and is able to learn which fractions are most important in determining complex membership. By fitting a multivariate Gaussian distribution to each cluster, a mean as well as a variance is assigned to each fraction of the migration profile. Thus, unlike other approaches, the GIP assigns a mean and variance to each fraction, allowing the model to learn for each cluster which parts of the migration pattern are determinant in assigning cluster membership. Furthermore, we use a bootstrapping workflow to identify clusters that likely represent actual complexes, and score proteins to prioritize actual interactors over spuriously comigrating proteins. To benchmark the performance of our approach and compare it to other state-of-the-art tools, we apply it to a set of publicly available human fibroblast complexome profiles. Additionally, we demonstrate its ability to identify novel interactions in less well-studied species by applying it to study the complexome of the transmissible forms of the deadliest human parasite causing malaria:*Plasmodium falciparum*.

## Methods

### Gaussian Interaction Profiler Workflow

#### Initial Clustering

The Gaussian Interaction Profiler takes as input a single complexome profiling data set, consisting of a series of abundance or intensity values that represent the fractions of the migration pattern for each detected protein. Any type of protein quantification metric that accurately reflects the relative abundance differences between fractions and proteins is suitable, as the GIP scales these abundance values to “*z*-scores” separately per protein such that each protein feature’s abundance values have a mean of 0 while the standard deviation is equal to 1. After scaling of the complexome profiles, the initial clustering is performed on the protein migration patterns, using a Gaussian mixture modeling (GMM) implementation that is fit using an expect-maximization (EM) algorithm, implemented in scikit-learn.^15^ Rather than determining pairwise similarity between two peaks in the migration pattern or the complete migration patterns of two proteins, GMM fits a multivariate Gaussian distribution to describe each cluster’s migration profile, where the number of dimensions is equal to the number of fractions of the complexome profiling sample. During model fitting with EM, the Gaussian mixture model parameters are determined while, simultaneously, proteins are assigned to these clusters based on how well their migration fits with its profile. This approach, unlike other methods, allows the model to learn the variation in each fraction, effectively learning which fractions are important to determine membership of each cluster. The number of clusters can be specified as a fraction of the number of protein features in the input. The result of the initial clustering is an assignment of all proteins to one of the clusters, and two vectors describing the Gaussian distribution of each cluster, containing the means and variances for each fraction, respectively.

#### Bootstrapping

After the initial clustering stage, a bootstrapping approach is used to estimate the stability of the clusters and determine the consistency with which each protein is part of a cluster, using a bootstrapping approach with a resampling strategy without replacement as introduced by Hennig.^16^ For a given number of iterations a resampled data set is generated by randomly sampling from the fractions. By default, a subset of half the total number of fractions in the complexome profiles is randomly selected without replacement, forming the resampled data set for the current bootstrap iteration. This data set is then clustered using Gaussian mixture modeling as described in the previous section. After every bootstrap iteration, the similarity of each bootstrapped cluster to every original cluster is determined using the Jaccard index,^16^ after which the bootstrapped clusters that are the closest match with each original cluster are selected. From each bootstrapping iteration, the similarities between each original cluster and its closest match as well as the members of these closest matching clusters are stored. After all bootstrapping iterations are completed the stability of each original cluster is determined by taking the average similarity between the original clusters and the best matching cluster in each of the bootstrap iterations. Additionally, the frequency with which each original cluster member, i.e., each protein, is part of the most similar cluster over all the bootstrap iterations is computed to obtain a confidence score per protein.

#### Scoring Clusters and Proteins

After the initial clustering and the bootstrapping stage, the assigned cluster members and clusters are annotated with additional information. The total abundance across all fractions is determined using the log-transformed protein abundance or intensity values. The total log-transformed abundances for all proteins are expressed as *z*-scores, where the average total abundance across all proteins is equal to 0 and the standard deviation is equal to 1. Aside from the cluster stability derived from the bootstrapping process, the fraction with the highest mean scaled abundance value for each cluster is determined. Furthermore, using the total protein abundances as described above, the average protein abundance is computed for each cluster.

### Benchmarking GIP Workflow

#### Complexome Profiling Data

The complexome profiling data used in this study are publicly available data sets deposited in the CEDAR database for complexome profiling data (www3.cmbi.umcn.nl/cedar/).^17^ All used data sets are from human fibroblast cell lines. Detailed information on the data used and references are shown in Table 1.

**Table 1:** *Homo sapiens* Complexome Profiling Samples Used in This Study

**sample id**	**proteins**	**study id**	**refs**
CRS17	2680	CRX8	(18)
CRS22	3098	CRX9	(19)
CRS23	3264		
CRS24	3173		
CRS25	2997		
CRS48	3081	CRX15	(20)
CRS50	4160	CRX17	(21)
CRS86	3031	CRX22	(22)

#### Reference Complexes and Evaluation Metrics

To evaluate the performance of the GIP, we used a set of reference protein complexes from CORUM v3.0.^23^ To determine the degree to which all tested clustering algorithms were able to recover the complexes detected, we determined the set of proteins that were detected in at least one of the fractions of each included human complexome profiling data set. The set of reference complexes were filtered such that only proteins that are in this set of reliably detected proteins were included. After this protein-filtering step, any complexes that no longer consist of more than one distinct protein were removed from the reference set.

#### Other Clustering Tools

Hierarchical clustering of the complexome profiles was performed using the python fastcluster package v1.2.6,^24^ using the Pearson correlation as similarity metric between protein migration patterns. To achieve clustering results that are comparable to other approaches, and to enable evaluation of the clustering results by comparing to a set of reference complexes, we used cut-points in the resulting tree-structure to achieve partitioning of the proteins into discrete clusters. To determine the optimal cut-point value and linkage method we performed clustering using a range of cut-points and the “single”, “average” and “complete” linkage methods. The results were evaluated as described above and are shown in Supporting Information Figure S1. The cut-point and linkage method resulting in the highest performance (average linkage, cut-point: 0.07) were used for all following analyses and comparisons in this study.

The ClusterOne software v1.0^25^ was applied to pairwise Pearson correlations computed between the migration patterns of all protein pairs. ClusterOne expects as input a set of weighted edges (interaction scores) connecting a network of nodes (proteins), which does not need to be fully connected, and a range of correlation score thresholds for inclusion of an edge in the algorithm input was tested. Inclusion of edges with a correlation score of 0.96 or higher resulted in the best performance. The optimal set of parameters was determined by performing clustering on one of the human data sets using a wide range of values for ClusterOne’s overlap, density, haircut, and penalty parameters. As the penalty parameter had minimal effect on performance, it was kept at its default value of 2. The results are shown in Supporting Information Figure S2. The parameter values that resulted in the highest MMR, and that were used for following analysis and comparisons, were as follows: cutoff = 0.96, density = 0.8, haircut = 0.8, overlap = 0.9.

The ComplexFinder pipeline^7^ was used in the mode that runs without reference database, to ensure fair comparison with the other clustering tools used in this project. This skips training of a classifier to predict interactions, but still performs clustering of the data using dimension reduction with UMAP^26^ followed by the hdbscan^27^ cluster algorithm. It can then base the dimension reduction and clustering on the raw protein migration pattern abundances. Alternatively, clustering can be based on a combined score of metrics computed from the migration patterns. Additionally, the hdbscan has a minimum cluster size and a “min_samples” parameter, which were also varied to explore their effect on performance. Using the raw protein abundances as input for UMAP, using minimum cluster size of 2 and a min_samples value of 1 resulted in the overall best performance, and were used for all analyses and comparisons in this study.

#### Comparing the Performance of Clustering Tools

To determine the agreement between the clustering results and the processed reference complexes, we use the maximum matching ratio (MMR), as described by Nepusz et al.^25^ As some of the tested clustering methods can produce singleton clusters containing only a single protein, these were not considered in determining MMR. All eight human samples were separately clustered using the GIP clustering workflow, hierarchical clustering, ClusterOne,^25^ and ComplexFinder.^7^ Each clustering method was run using the optimized (hyper-)parameters as described in the previous section. The clustering results were evaluated by comparing them to the processed CORUM reference complexes,^23^ resulting in 8 MMR values per clustering approach, each from one of the human complexome profiling samples. To determine the performance of each clustering tool on complexes of various sizes, the set of reference complexes were divided into four categories containing complexes with size 2, 3, 4, and 5 or more subunits respectively. The performance of each clustering algorithm for each category was computed separately as described earlier.

#### Cluster and Protein Prioritization Analysis

To determine the degree to which the cluster metrics computed by the GIP are able to prioritize clusters representing protein complexes over other clusters, the distribution of these scores for clusters corresponding to reference complexes was compared to the distribution of scores for all clusters. All eight human complexome profiles were analyzed separately with the GIP. The clusters resulting from separate clustering of each of the eight human complexome profiles were pooled for this analysis. All clusters with an overlap of at least 0.75 with one of the reference complexes were annotated as reference complexes. To determine the degree to which a metric prioritizes known complexes, a threshold value was set and only the complexes that met this threshold were retained. After filtering using one of the metrics, the fraction of retained reference complexes was compared to the overall fraction of retained complexes. This fraction was computed for a range of threshold values covering the distribution of each metric.

The clusters that matched with one of the reference complexes were used to perform a similar analysis with the protein-level abundance and frequency metrics. Since there is expected to be variation in abundance and stability between clusters, we performed a transformation of the scores that only compares these metrics within each cluster. To do this, the members of each cluster are ranked on the given metric. The center of the position that each score takes in the ranked list is computed, on a scale between 0 and 1, with 1 being on the top of the list. For example, if a cluster contains 10 proteins, each with a different abundance value, the protein with the highest abundance gets a value of 0.95, while the protein with the second highest abundance value gets assigned a value of 0.85, etc. These rank position scores are then analyzed as previously described for the cluster-based metric to determine the degree to which these metrics prioritize true interactors over noninteracting proteins within clusters.

### Complexome Profiling of *P. falciparum*

#### Parasite Culture and Gametocyte Induction

A *P. falciparum* NF54/iGP2^28^ line was cultured using standard techniques in RPMI medium supplemented with 10% human serum at 5% hematocrit and 2.5 mM D-(+)-glucosamine hydrochloride (Sigma #1514). To induce gametocytogenesis, glucosamine was excluded from the medium. ABS elimination was achieved by treating the culture with 50 mM N-acetylglucosamine from days 4–8 postinduction. On day 14 postinduction, mature gametocytes were magnetically purified as described previously.^29^

#### Sample Preparation

Infected red blood cells containing gametocytes were treated with 0.05% (w/v) saponin in phosphate-buffered saline (PBS; pH 7.4) solution for 10 min to remove host material. Following a centrifugation step at 3000*g* for 5 min, the resulting parasite pellets were resuspended and washed twice in a solution containing 250 mM sucrose, 10 mM HEPES, 1 mM EDTA, and 1× cOmplete EDTA-free Protease Inhibitor Cocktail (Sigma) at pH 7.4. The washed parasite pellets were transferred to a prechilled cell disruption vessel (#4639 Parr Instrument Company) and pressurized with nitrogen gas at 1500 psi and then left to equilibrate for 10 min. Parasite cells were subjected to nitrogen cavitation through a controlled release, resulting in cell shearing and yielding the parasite lysate. To reduce sample complexity and potentially obtain subcellular fractions, the lysate was layered on top of a discontinuous gradient comprising 25, 35, 45, and 60% sucrose layers. Ultracentrifugation was conducted at 100,000*g* for 1 h. Postcentrifugation, samples were collected from visible bands at the interfaces of the sucrose layers, yielding three distinct fractions: 25–35, 35–45, and 45–60%.

#### High-Resolution Clear Native Electrophoresis (hrCNE)

Fractions obtained from the sucrose gradient ultracentrifugation were resuspended in a buffer containing 50 mM sodium chloride, 50 mM imidazole/HCl, 2 mM 6-aminohexanoic acid, and 1 mM EDTA (pH 7). For solubilization, digitonin was added at a 6:1 protein-to-detergent ratio. Following solubilization, insoluble components were pelleted by centrifugation at 22,000*g* for 30 min at 4 °C. The solubilized protein samples were then subjected to hrCNE on a 3–16% gradient polyacrylamide gel, based on the method described by Wittig et al.^30^ An anode buffer containing 25 mM imidazole/HCl (pH 7.0) and a cathode buffer containing 50 mM Tricine, 7.5 mM imidazole (pH 7.0), 0.05% deoxycholate (DOC), and 0.02% dodecyl-maltoside (DDM) were used for electrophoresis.

After electrophoresis, gels were fixed, stained with Coomassie-blue G-250, and destained. Gels were imaged using an ImageScanner III (GE Healthcare) to guide slicing. Adapting from Heide et al.,^3^ gel lanes were sectioned into 60 uniform slices, subdivided, and transferred to a filter microplate. After Coomassie dye removal, cysteine reduction and alkylation were conducted. Gel pieces were dehydrated, treated with sequencing-grade trypsin (Promega), and incubated overnight at 37 °C. Peptides were recovered into 96-well PCR microplates, eluted, SpeedVac-dried, reconstituted in a 5% ACN, 0.5% FA solution, and stored at −20 °C until subsequent MS analysis.

#### Mass Spectrometry

Peptides were chromatographically separated and analyzed on a Q Exactive mass spectrometer with an Easy nLC1000 system (Thermo Fisher Scientific). Using a PicoTip emitter column (New Objective) filled with ReproSil-Pur C18-AQ beads (Dr. Maisch GmbH), peptides underwent a gradient elution from 5–35% ACN, 0.1% FA over 30 min, followed by a ramp to 80% ACN, 0.1% FA in 5 min. The mass spectrometer was set in positive mode, autoswitching between MS and MS/MS for the top 20 precursor ions. Key parameters included a full-scan MS range of 400–1400 *m*/*z* at 70,000 m/Δm resolution and MS/MS parameters of 17,500 m/Δm resolution with 4.0 Th isolation window, targeting precursor ions of charge *z* = 2 and 3. Internal calibration employed a lock mass ion at *m*/*z* = 445.12. For a detailed methodology, we refer to Evers et al.^31^

#### Protein Identification

Raw MS data from all slices were processed using MaxQuant (v1.5.0.25).^32^ Peptide spectra were searched against a *P. falciparum* reference proteome (isolate 3D7, July 2022, from uniprot.org) including common contaminants like BSA, pig trypsin and human keratins. Noteworthy parameters included: variable modifications of N-term acetylation and methionine oxidation; up to two missed trypsin cleavages; cysteine carbamidomethylation as a fixed modification; a 2 min window and unidentified features matching; and a 1% FDR target using the target-decoy approach. Label-free quantification was employed with iBAQ values. Any proteins for which there was no direct MS/MS evidence in one of the complexome profiling samples discussed in this paper were discarded from the results. The resulting complexome profiling data have been deposited in the CEDAR database (https://www3.cmbi.umcn.nl/cedar/browse/experiments/CRX48)^17^ profiles. The mass spectrometry proteomics data have been deposited to the ProteomeXchange Consortium via the PRIDE^33^ partner repository with the data set identifier PXD050751.

#### Mass Calibration

In order to match the hrCN fractions to estimated molecular masses, bovine heart mitochondria were solubilized under the same conditions as the samples and ran alongside on the same gel. Bands made visible through coomassie brilliant blue staining could be matched to mitochondrial OXPHOS complexes with known mass and corresponding slices of the gel to create a calibration curve as described previously.^34^ The mass calibration was performed using the masses of the following bovine mitochondrial OXPHOS complexes: CII (123 kDa); CIV (215 kDa); CIII (485 kDa); CV (700 kDa); CI (1000 kDa); supercomplex I–III (S0, 1500 kDa); supercomplex I–III–IV (S1, 1700 kDa); supercomplex I–III–IV2 (S2, 1900 kDa) (Supporting Information File 2.).

## Results

### Gaussian Interaction Profiler Tool Overview

**Figure 1:**
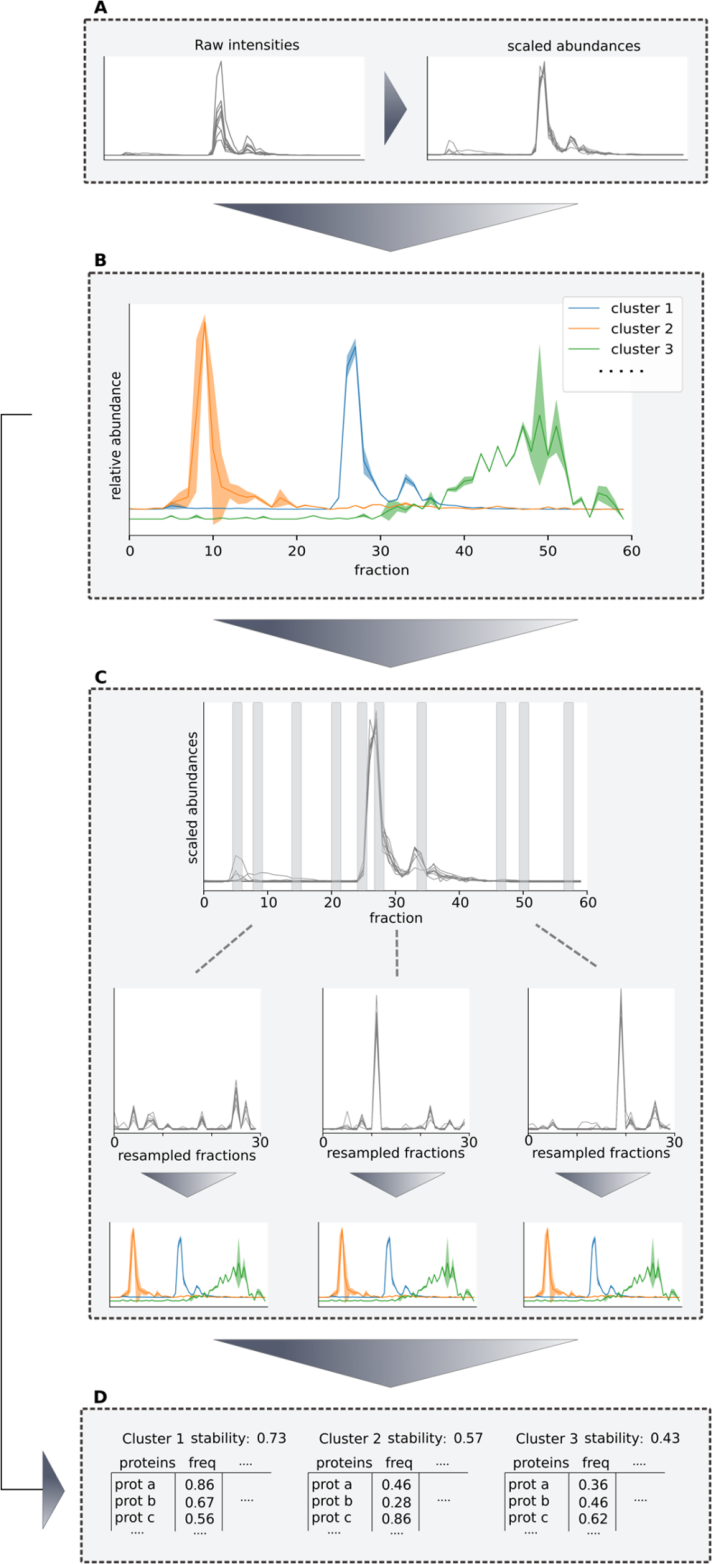
Overview of the GIP clustering workflow. (A) The input of the workflow is a single complexome profiling sample, with an abundance or intensity value for each fraction per protein, representing its migration pattern. Before clustering, each migration pattern is scaled to *z*-scored relative abundances. (B) The protein migration patterns are partitioned into a predetermined number of clusters by fitting a Gaussian mixture model using an expectation-maximization approach. (C) After the initial clustering stage, a bootstrapping approach is used, repeatedly clustering resampled data sets. (D) The results from the original clustering and the bootstrapping are combined to produce resulting clusters with cluster-level as well as protein-level reliability metrics derived from the bootstrapping.

Our Gaussian Interaction Profiler (GIP) is a Gaussian Mixture modeling-based workflow that performs clustering on a complexome profile to systematically identify candidate protein complexes in an automated manner. It aims to overcome some of the challenges with identification of novel candidate protein complexes from complexome profiling data by prioritizing clusters that represent true interactions as well as prioritizing actual interactors among the clustered proteins. An overview of the the GIP workflow is shown in Figure 1. The input of our method is a single complexome profile, consisting of a protein migration pattern for each detected protein. A migration pattern is a series of abundance or intensity values, one for each of the fractions in which the sample was separated. Prior to the analysis, each protein migration pattern is scaled to have a mean of 0 and a standard deviation of 1, often referred to as “*z*-scores”. After scaling, a Gaussian mixture model is fit, using an expectation-maximization (EM) approach.^15,35^ During this process, the EM algorithm aims to find an optimal assignment of all detected protein features to a predetermined number of clusters, which are each modeled as a multivariate Gaussian distribution. Once the mixture model is fit, each protein has been assigned to a cluster, and each cluster is described by the means and (co–) variances describing the “migration profile” across the fractions for each cluster.

After initial clustering of the complexome profile, a bootstrapping approach is used to determine how strongly each cluster is represented in the data, as well as the consistency with which each protein is part of a cluster. By repeatedly sampling fractions from each migration pattern and then repeating the clustering procedure on the resampled data, multiple rounds of clustering results are generated. These results are then compared to the initial clustering results to determine the degree to which each original cluster is recovered in the bootstrapped results, as described in Hennig et al.^16^ Additionally, the bootstrapped results are used to determine the frequency with which each originally clustered protein is assigned to the same group of proteins during bootstrapping. The resulting cluster stability and protein cluster frequency metrics are reported in the output and can be used to prioritize likely relevant clusters and cluster members. In addition to these bootstrapping-based metrics, several other cluster and cluster member features are computed. First the relative abundance of each protein is determined prior to normalization of the data, which is used to compute the average abundance for each cluster. Additionally, the average mutual information between protein members of each cluster is computed. The complete GIP workflow is implemented as a user-friendly and flexible python (v3) package, available on github (github.com/joerivstrien/gip-bio).

### Benchmarking Recovery of the Human Complexome with the GIP

To determine the effectiveness of our approach in recovering protein complexes, we have applied our method to a set of eight publicly available human complexome profiles. These are all complexome profiles from human fibroblast cell lines, enriched for mitochondrial protein complexes. A detailed overview of the data sets used is available in Table 1. To evaluate the degree to which our approach is able to recover the complexes detected in these data, we use a reference set of known protein complexes from CORUM v3,^23^ from which we filtered out proteins and complexes that are not consistently detected in the selected complexome profiles.

**Figure 2:**
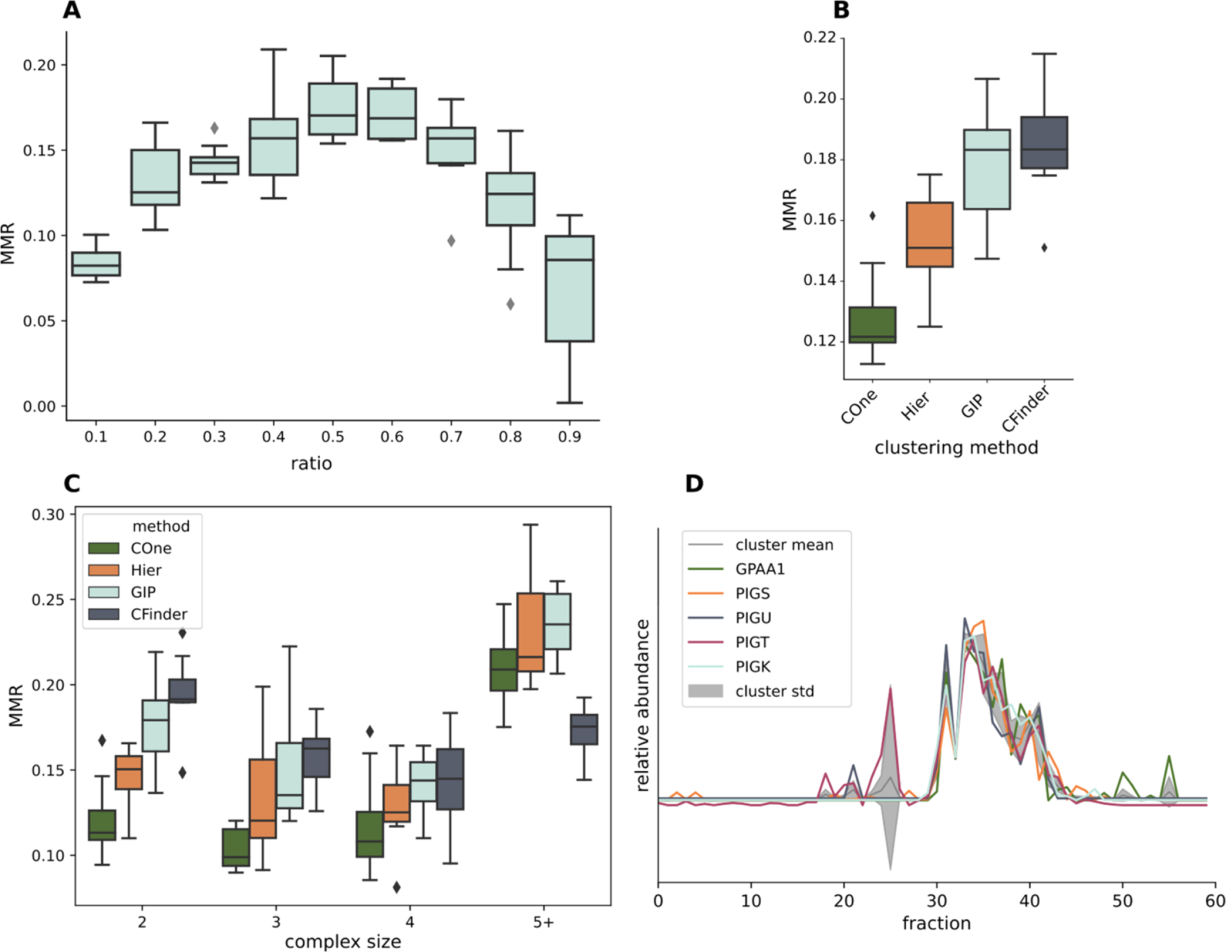
Benchmarking the performance of the GIP in recovering known protein complexes from human complexome profiles, compared to other methods used to cluster complexome profiling data. (A) The recovery of a set of CORUM reference complexes^23^ from eight human complexome profiles with the GIP, using a range of complex-to-protein ratios. As an example, a ratio of 0.5 means that the number of clusters used in Gaussian mixture modeling is equal to half of the number of proteins detected in the data set. The agreement between the clustering results and the reference complexes is determined using the maximum matching ratio.^25^ The boxes represent quartiles, whiskers extend to points within 1.5 times the interquartile range and any outliers are shown as diamonds. (B) the performance of the GIP compared to clusterOne (COne),^25^ hierarchical clustering (Hier)^24^ and ComplexFinder (CFinder)^7^ The boxes represent quartiles, whiskers extend to points within 1.5 times the interquartile range and any outliers are shown as diamonds. (C) The performance of each aforementioned clustering method, stratified by the size of the reference complexes. The boxes represent quartiles, whiskers extend to points within 1.5 times the interquartile range and any outliers are shown as diamonds. (D) Visualization of the migration patterns of clusters containing the complete GPI-anchor transamidase complex. The mean ± the variance for each fraction from the fit Gaussian mixture model are shown, as well as the migration of each of the cluster’s members. Even though some complex subunits show different migration in lower fractions, the GIP correctly clusters them together.

Gaussian mixture modeling requires specification of the number of clusters prior to clustering. Ideally, we would like to know the number of clusters that results in the optimal performance beforehand. Unfortunately, this is not feasible when clustering a new data set as the optimal number of clusters is very likely to vary depending on various features of the complexome profile under investigation. To overcome this, we assume that the optimal number of clusters is linearly related to the number of detected proteins in the data set. Thus, we attempted to determine an optimal ratio between the number of detected proteins and the number of clusters. The recovery of our reference set of protein complexes when clustering the human data sets, which range in size from 2680 to 4160 detected proteins, using various ratios is shown in Figure 2A. As expected, we found a relation between the cluster number ratio and the clustering performance, where a ratio of around 0.5–0.6 of the number of clusters relative to the number of detected proteins seems to result in good performance regardless of the data set size.

To compare the performance of the GIP to the state-of-the-art in complexome profiling analysis, we determined the performance of several other methods that are commonly used to cluster complexome profiles. To ensure a fair comparison, we compared our approach to state-of-the-art methods that, like our approach, do not rely on any external evidence or a reference set of known protein complexes: hierarchical clustering,^24^ ClusterOne^25^ and ComplexFinder.^7^ While ComplexFinder does have the option to use a set of reference interactions to generate improved interaction scores, we use it in the mode that does not rely on any external information. The optimal (hyper-)parameters of these tools were determined as detailed in the methods. The optimized performance of these tools compared to the GIP is shown in Figure 2B. ComplexFinder and GIP show similar performance, and significantly outperform ClusterOne and hierarchical clustering (Table S1, Supporting Information Figure S3).

To explore the performance of each clustering method on complexes of different sizes, the set of reference complexes was separated into four categories: protein complexes consisting of 2, 3, 4, and 5 or more (5+) different proteins. The performance of each tool on these separate categories is shown in Figure 2C. The GIP scores significantly higher than ClusterOne for all size categories (Table S1). The GIP outperforms hierarchical clustering for complexes of size 2, has a higher MMR in 7 out of 8 samples for complexes of size 3 and performs similarly for complexes consisting of four or more proteins. CFinder does not show significantly higher performance compared to the GIP in any size category and has the lowest performance out of all tested methods in complexes of at least five proteins (Figure 2C, Figure S3, Table S1). The total number of clusters generated by ClusterOne is higher than other approaches and the mean cluster size is smaller, which explains the relatively poor performance in recovery of larger protein complexes (Supporting Information Figure S4). As the GIP models each cluster as a multivariate Gaussian distribution, it allows certain fractions to have larger variance than others. This can help cluster complex members that comigrate in specific fractions of the migration pattern while allowing them to be divergent in others. An example of this is the GPI-anchor transamidase complex, consisting of five protein subunits, that posttranslationally attach GPI-anchors to proteins, which anchor these proteins to the cell surface.^36^ The full complex is completely captured in the human CRS24 sample with the GIP, even though two of its subunits (PIGT and GPAA1) show divergent migration to the other subunits in areas of their migration patterns not relevant to this complex (Figure 2D). ClusterOne does not cluster any of its subunits together and only part of the subunits are coclustered with hierarchical clustering (PIGS, PIGU, PIGK) and ComplexFinder (GPAA1, PIGS).

### Prioritizing Clusters

With complexome profiling, a large number of proteins are separated across a limited number of fractions, resulting in comigration of many proteins with similar mass that do not actually interact. In practice this means that when these data are clustered, many clusters do not represent actual complexes, and in clusters representing actual complexes not all clustered members are actual interactors. To overcome this, our approach computes several cluster metrics and clustered protein metrics, with the aim to simplify prioritization of likely biologically relevant clusters and interactors. The clustered protein metrics are the frequency with which each cluster member is part of their original cluster during bootstrapping and the protein relative abundance. The cluster-level metrics are the location of the fraction with the highest mean abundance value in the cluster migration pattern (“maxloc”), the bootstrapping-based cluster stability, the average mutual information between cluster members as well as the average relative abundance of the cluster members.

**Figure 3:**
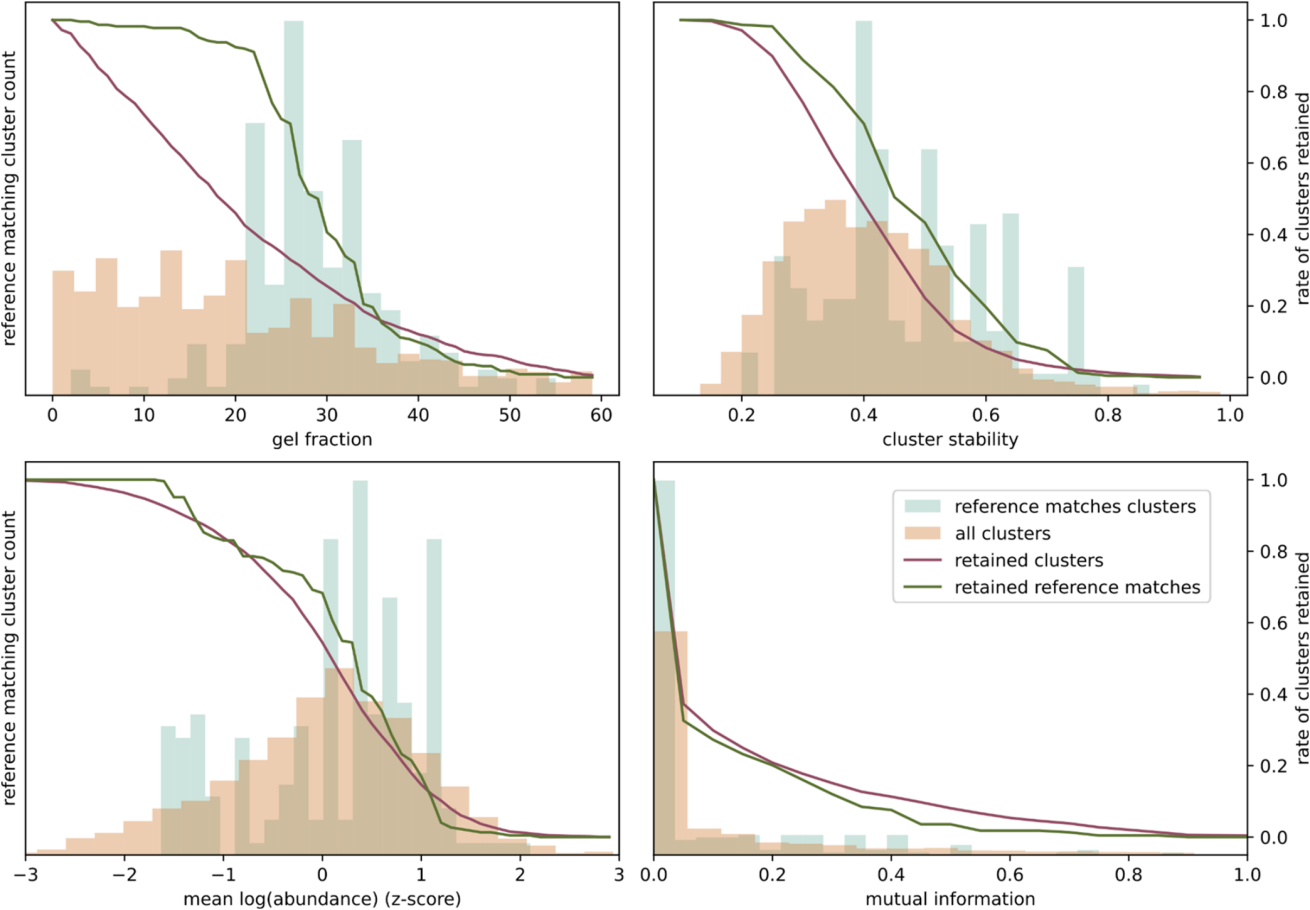
Visualizing the effectiveness of various cluster-level metrics in prioritizing complexes from the results of applying the GIP to the human complexome profiles. The bars show the distribution of each cluster metric as indicated on the *x*-axis for all identified clusters (beige) compared to that of the CORUM reference complexes (mint). The lines signify the effect of using the respective metrics as a cutoff filter on the retention of all clusters (red) as compared to the reference complexes (green). The line *y*-axis values (right side) correspond to the fraction of retained clusters after removing any clusters with a metric value equal or lower to the value on the *x*-axis.

To determine the effectiveness of the cluster-level metrics to identify clusters that represent actual complexes, we examined the distribution of these metrics across clusters that represent known complexes and compared these to the overall distribution (Figure 3). Additionally, this figure shows the effect of filtering the clusters with each metric on the retention of known protein complexes. We can see that known protein complexes tend to score at the higher end of the overall distribution of both the “maxloc” and the bootstrapped cluster stability scores, and that filtering the clusters on these metrics results in enrichment of biologically relevant clusters. For example, removing clusters with a maxloc below 10 would reduce the total number of resulting clusters by 27%, while only losing 1.7% of the clusters that match a known reference complex. While the mean cluster abundance does not show as clear of a pattern, none of the known complexes have an abundance *z*-score below −2. To further investigate whether the identification of protein complexes with complexome profiling and the GIP is biased toward complexes with higher abundance, we have determined the recovery of each detected CORUM reference complex in each sample by a GIP result cluster. There is no clear relation between the recovery of a reference complex and their abundance in the investigated complexome profiling sample (Supporting Information Figure S5). The average mutual information does not show clear enrichment of known reference complexes and thus seems a poor metric to prioritize clusters that represent actual complexes.

**Figure 4:**
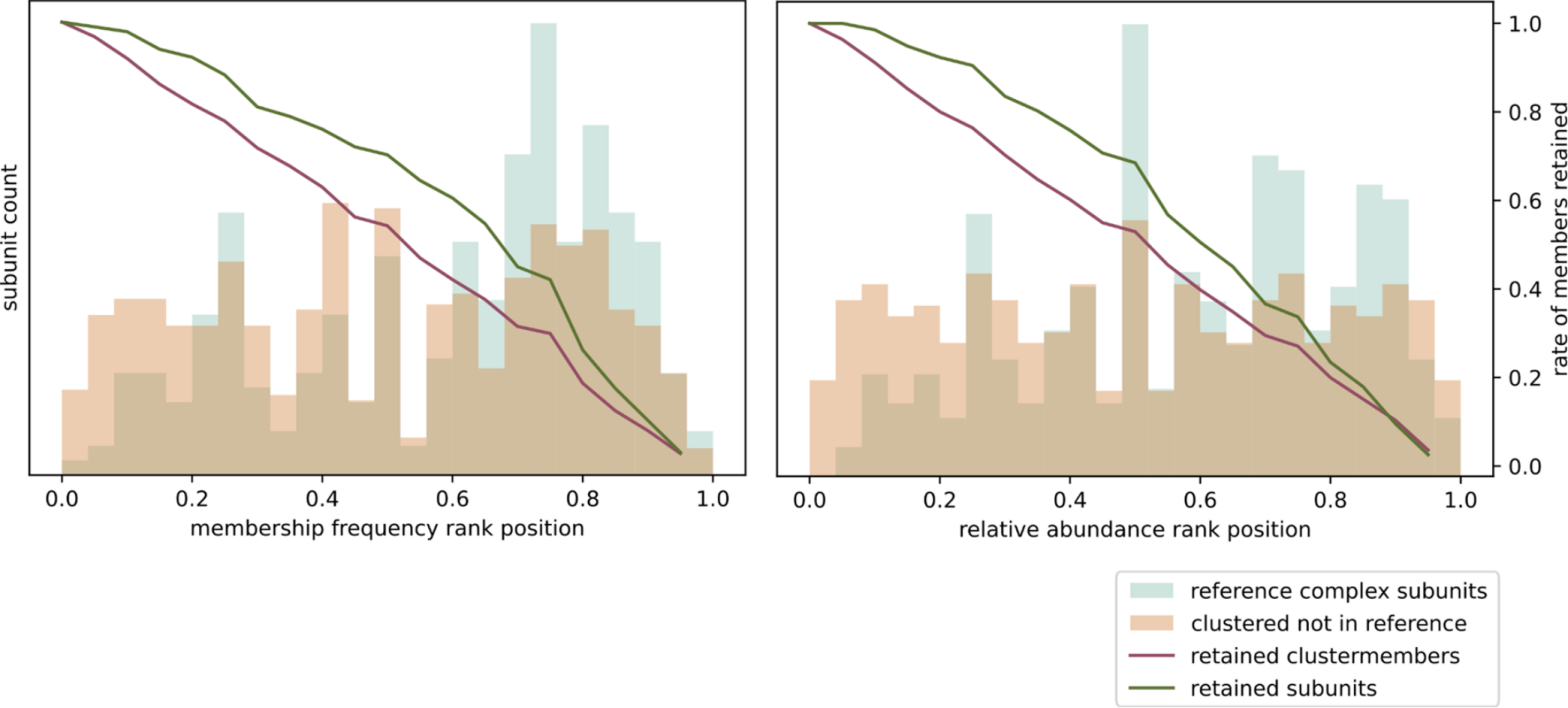
Visualizing the effectiveness of the protein frequency and abundance metrics in prioritizing complex members over other clustered proteins. All protein members from the GMM-workflow resulting clusters matching one of the reference complexes were used for this analysis. The rank position of each cluster member when sorting them on either their abundance or frequency was computed. These rank positions were used to determine the degree to which these metrics are able to prioritize known interactors over other clustered proteins in each of the tested clusters. The bars show the distribution of the binned rank positions for all identified clustered proteins (salmon) compared to those known to be part of the respective CORUM reference complexes (mint). The lines signify the effect of using the respective metrics as a cutoff filter on the retention of all clustered proteins (red) as compared to known interactors (green). The line *y*-axis values (right side) correspond to the fraction of retained proteins after removing any proteins with a metric value equal or lower to the value on the *x*-axis.

In a similar fashion, to determine the effectiveness of the clustered member-level metrics in prioritizing true complex subunits over spuriously comigrating proteins, we investigated the distribution of the cluster frequency and protein abundance metrics for proteins in clusters that correspond with one of the reference complexes. There is variation in these metrics between clusters, i.e., some clusters representing complexes will be more abundant or stable than others. Therefore, we focus on the rank position of each protein relative to the other clustered proteins, rather than using the absolute values for these metrics. The results of this analysis are shown in Figure 4. As expected, we can see that known subunits tend to score higher relative to the other proteins of their cluster, both in terms of frequency and abundance.

### Applying the GIP to Complexome Profiles from Malaria Parasites

To explore its utility, we applied the GIP to three not previously investigated complexome profiles derived from the sexual stages of *P. falciparum*, the deadliest human parasite species causing malaria. Briefly, whole parasite cells were isolated and subjected to nitrogen cavitation to free cellular content and homogenize the sample. To decrease sample complexity, the homogenate was consequently separated through ultracentrifugation using a sucrose gradient. The three resulting fractions were separately resolved by high-resolution clear native electrophoresis followed by mass spectrometry and complexome profiling analysis. This resulted in three complexome profiles, containing the migration profiles of 1906 unique *P. falciparum* proteins across 60 gel slices. Given that no well-curated set of protein complexes expected to be present in the *P. falciparum* gametocyte life stage exists as of yet, we are not able to systematically evaluate the GIP’s performance in these samples. However, as this is not the first study applying complexome profiling to *P. falciparum* samples, we examined whether the GIP is able to reconstitute previously described protein assemblies. We found that the GIP correctly clusters many of the components of described complexes such as mitochondrial respiratory complexes, signal peptidase complex, signal recognition particle, translation initiation complex, proteasome, and V-type ATPase (Tables 2, S2). In the case of large protein assemblies, the clusters often did not represent the full complex but instead the full complex was split across multiple clusters, such as the V-type ATPase complex that is known to fall apart into at least two subcomplexes after native electrophoresis separation.^37^ Taken together, the GIP enabled us to reconstitute various complexes including conserved eukaryotic complexes, complexes derived from manual analysis,^31^ and complexes revealed by our comparative clustering approach^38^ that integrates additional data alongside complexome profiles.

**Table 2:** Complexes Identified in Complexome Profiles of *P. falciparum*^a^

**complex**	**sucrose gradient subsample %**	**clust_id**	**size**	**maxloc**	**stability**	**mean abundance (***z*-score)
signal peptidase complex	35–45	54	6	15	0.46	0.89
translation initiation factor 3	35–45	232	5	34	0.41	–0.23
V-type ATPase	25–35	121	8	34	0.59	1.30
V-type ATPase	25–35	578	7	26	0.58	1.35
20S proteasome	35–45	878	12	28	0.51	0.04
20S proteasome	45–60	797	6	28	0.41	0.30
26S proteasome	35–45	140	4	34	0.41	–0.60
26S proteasome	45–60	856	8	33	0.33	–0.37
respiratory chain complex III	35–45	962	5	29	0.43	2.13
respiratory chain complex III	35–45	28	9	29	0.45	1.50
respiratory chain complex III	45–60	23	12	30	0.70	1.61
respiratory chain complex IV	35–45	743	9	26	0.41	1.08
respiratory chain complex IV	35–45	41	11	27	0.58	1.44
respiratory chain complex IV	45–60	54	23	27	0.55	1.06
ATP synthase	35–45	21	7	41	0.78	–0.08
ATP synthase	45–60	23	12	30	0.70	1.61
Pfs47 complex	25–35	303	2	13	0.82	1.45
Pfs47 complex	35–45	56	3	12	0.55	0.48
mitochondrial carrier complex	35–45	158	4	9	0.41	0.67
concavalin A complex	45–60	299	2	24	0.78	1.34
calcium binding interaction	45–60	894	2	22	0.87	1.67

a This table contains previously described complexes that were represented by one or more clusters resulting from application of GIP to *P. falciparum* complexome profiles. In addition to the previously described complexes the table contains several clusters containing putative novel protein complexes. The Pfs47 clusters contain a putative protein complex consisting of Pfs47 interacting with another female gametocyte-specific protein. The Mitochondrial carrier cluster contains a putative protein complex consisting of carrier proteins associated with the inner mitochondrial membrane. The Concavalin A cluster contains a putative interaction between two proteins from the concanavalin A-like lectin/glucanase superfamily. The calcium-binding cluster contains a putative interaction between two proteins from a family of PCM4-related calcium/calmodulin binding membrane proteins.

### Investigation of High-Scoring Clusters for Potential New Interactions

Further examining the list of generated clusters, we also identified a number of smaller clusters that have high bootstrapping stability. Below we highlight a selection based on their biological plausibility and potential interest for the malaria research community.

#### Pfs47 Associates with a Female Gametocyte-Specific Protein

**Figure 5:**
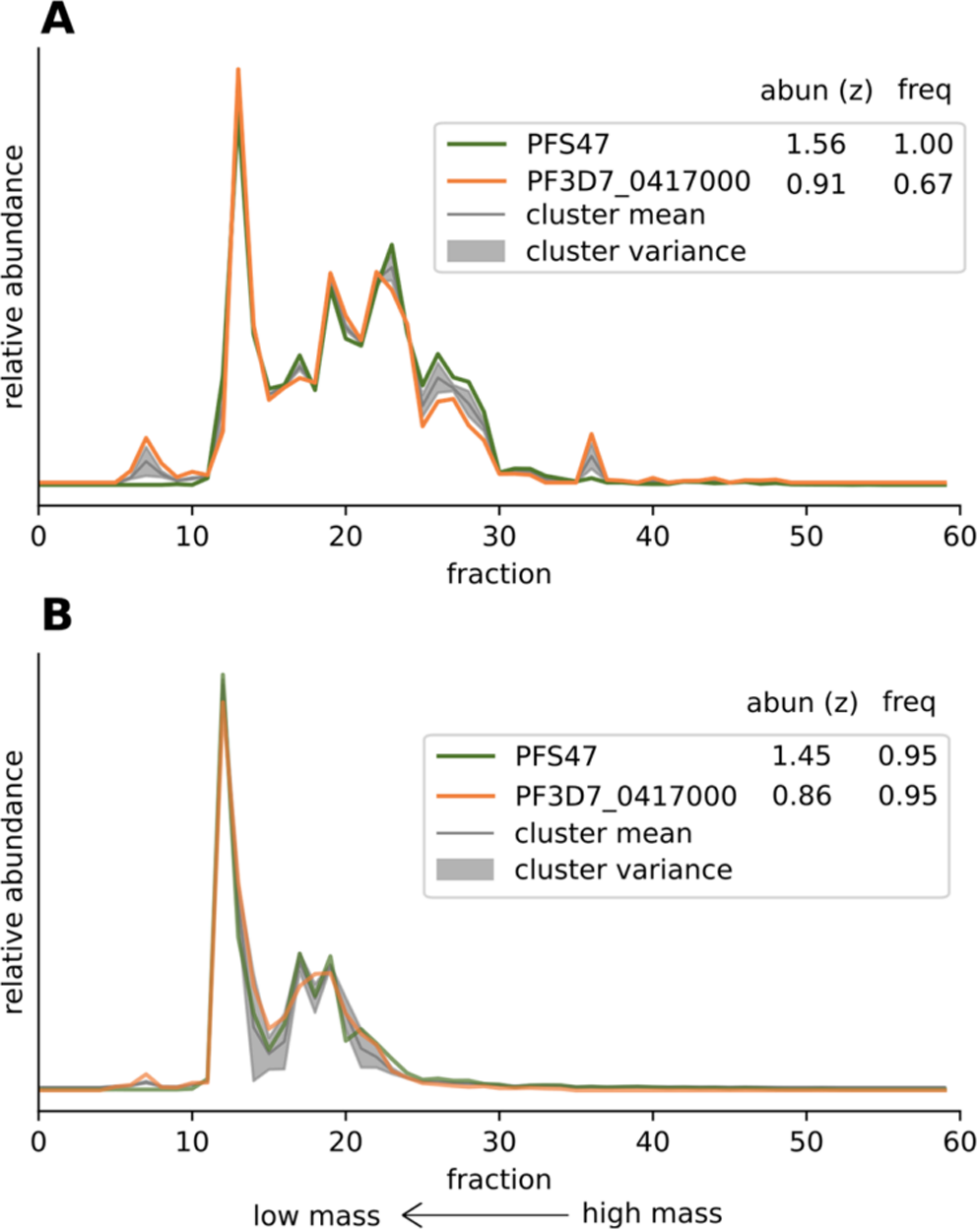
Visualization of the migration patterns of clusters containing Pfs47. The mean ± the variance for each fraction from the fit Gaussian mixture model are shown, as well as the migration of each of the cluster members. The legend shows the clustered proteins, with their total log-transformed abundance across all fractions shown as *z*-scores, as well as their cluster frequency score. (A) Cluster 303 from subsample 25–35% (B) Cluster 56 from subsample 35–45%.

Pfs47 is a member of the 6-Cys protein family that is specific to the apicomplexan phylum and known to play a critical role in the host transition from human to the mosquito.^39^ Pfs47 is a female gametocyte-specific surface protein and has garnered attention due to its essential role in evading the mosquito immune system and as a validated target for transmission blocking interventions and vaccine development.^40−42^ While conserved among all *Plasmodium spp*., the sequences are highly diverse between species. Its paralog, Pfs48/45 is another vaccine candidate and known to interact with at least one other potent transmission blocking target,^43^ although their identity is still controversial,^44,45^ but to-date no proteins that interact with Pfs47 had been identified. We find that Pfs47 (51 kDa) clusters together with PF3D7_0417000 (33 kDa) in two of our subsamples (Figure 5). The main peak is shared in fraction 12 which corresponds to an estimated molecular mass of 109 kDa. This does not match 84 kDa, which is the sum of both proteins. This could be indicative of stable lipid interactions, posttranslational modifications or differing stoichiometry with for example two copies of PF3D7_0417000 for one copy of Pfs47. Besides the main peak, the proteins also comigrate at higher masses, particularly in the 25–35% subsample, which could be indicative of additional complex members or multimeric states. The fact that the migration shift between the two subsamples is identical between the two proteins further supports that this cluster represents a real interaction rather than spurious comigration. Like Pfs47, PF3D7_0417000 is far more abundantly transcribed in female than male gametocytes (>22-fold).^46^ The protein does not contain any recognizable functional domains except for an N-terminal transmembrane signaling sequence (likelihood 0.6),^47^ making it a protein that is, like Pfs47, potentially exported. Much like Pfs47, the protein is conserved across all sequenced *Plasmodium* species.^39^ Due to the high medical relevance, we find this putative interaction warrants further exploration potentially to aid currently ongoing vaccine and antibody design efforts, particularly in the light of the more conserved protein sequence and thus potential epitopes for an effective transmission blocking vaccine.^48^

#### Potential New Complex of the Inner Mitochondrial Membrane

**Figure 6:**
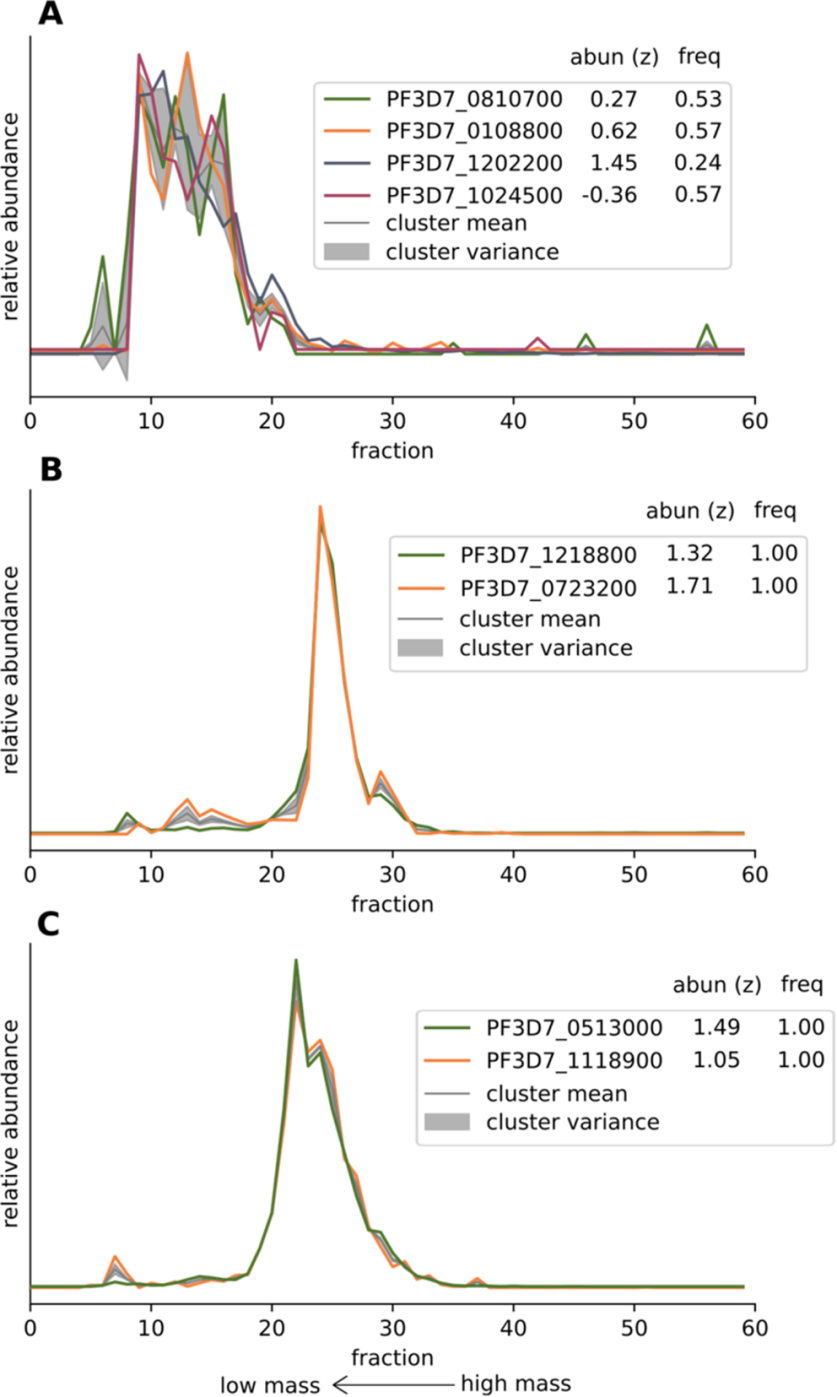
Visualization of the migration patterns of various clusters of interest. The mean ± the variance for each fraction from the fit Gaussian mixture model and the migration of each of the cluster’s members are shown. The legend shows the identifiers of the clustered proteins with their total log-transformed abundance across all fractions shown as *z*-scores as well as their cluster frequency score. (A) Cluster 158 from subsample 35–45%, which contains putative carrier proteins associated with the inner mitochondrial membrane. (B) Cluster 299 from subsample 45–60%, which contains two members of the concanavalin A-like lectin/glucanase domain superfamily. (C) Cluster 894 from subsample 45–60%, which contains two members from a family of PCM4-related calcium/calmodulin binding membrane proteins.

We also find a cluster of four proteins that could represent an intriguing interaction on the inner mitochondrial membrane (Figure 6A). It contains two proteins of the mitochondrial carrier family, a putative phosphate carrier (PfMPC, PF3D7_1202200, 36 kDa), and an unusual apicomplexan-specific mitochondrial carrier (PfAMC1, PF3D7_0108800, 42 kDa).^49^ A third protein of the cluster, PF3D7_1024500 (31 kDa), is predicted to be mitochondrial.^50^ Using HHpred^51^ we detected homology to two small mitochondrial proteins: the translocase of the inner mitochondrial membrane protein TIM21 and the cytochrome oxidase complex IV assembly protein COA1 that are homologues of each other.^52^ Just like most mitochondrial carrier family members, TIM21 and COA1 are located in the inner mitochondrial membrane, where TIM21 plays a role in connecting the translocases of the inner and outer mitochondrial membrane (TIM and TOM, respectively). In addition, TIM21 was described to couple TIM to OXPHOS supercomplexes,^53^ while COA1 has been shown to play a role in both assembly of CI and CIV.^54^ Within the context of the association between two mitochondrial carriers and this TIM21/COA1 homologue, it is interesting to note that recent studies have highlighted an emerging link between several mitochondrial carriers and CIV assembly.^55^ The fourth member of this cluster is PF3D7_0810700 (kDa), a small, uncharacterized protein that is conserved in *Plasmodium* species but has no homology to annotated proteins nor contains any functional domains other than a transmembrane helix. The four proteins share a relatively broad migration pattern from fraction 10 to fraction 17, which corresponds to an apparent molecular mass of 86–182 kDa and a further shared peak at fraction 20, corresponding to 251 kDa. This relatively broad migration pattern makes it challenging to clarify exact composition or stoichiometry of the putative complex. The sum of the proposed members, 126 kDa, falls within the range of the apparent molecular mass of the complex. The broad peak might represent an unstable complex in various disassembly states with the final shared peak at 251 kDa representing the intact (dimeric) complex. While the diverse composition makes it hard to assign a likely function of this complex in transport or respiratory chain assembly or functioning, there is precedent for divergent mitochondrial complexes in malaria parasites.^31^

#### Association of Lectin/Glucanase Domain Proteins

The two members from the Concanavalin A-like lectin/glucanase domain superfamily in *Plasmodium*, PF3D7_1218800 (40 kDa) and PF3D7_0723200 (41 kDa), cluster together and share a main peak in fraction 24, corresponding to 384 kDa, clearly above their monomeric mass (Figure 6B). Proteomic evidence suggests gametocyte specificity for both proteins.^56^ Since they have similar mass and high sequence similarity, it is unclear whether this constitutes an interaction between these two proteins, whether they form homomers or whether they, through their lectin-domain, simply bind carbohydrates to a similar degree resulting in migration above their monomeric mass.

#### Putative Association of Two Calcium-Binding Proteins

We identify two proteins, PF3D7_0513000 (31 kDa) and PF3D7_1118900 (29 kDa), that are conserved in Apicomplexa and consistently cluster together (Figure 6C). They share a main peak in fraction 22 which corresponds to 310 kDa, clearly above the mass of a putative heterodimer. Whether the migration at this mass is the result of interaction with other proteins not detected with our approach or presence of more copies of either protein is not clear at this point. Both proteins have so far been only detected in gametocytes, are part of the same family of PCM4-related calcium/calmodulin binding membrane proteins and harbor a signal peptide. Given their shared features and gametocyte specificity as well as the importance of calcium signaling for gametocyte biology,^56−58^ we think this putative interaction warrants further investigation.

## Discussion

With the GIP workflow we have developed an approach that enables identification of protein complexes using only complexome profiling data, without requiring additional evidence of interaction or a reference of known protein complexes. While gauging the accuracy of this capacity is challenging without additional experimental data, we have demonstrated that the GIP is consistently among the top performing methods in recovering known protein complexes for complexes of various sizes from a single complexome profile. Given the unsupervised nature of the algorithm used in the GIP, we expect it to perform similarly on complexome profiles generated with slightly different experimental approaches or representing species other than those studied in this work. However, given the relatively small number of data sets used to evaluate its performance, this work should be considered a proof-of-concept of its potential utility, rather than a thorough benchmark of its performance.

Other methods that do integrate multiple complexome profiling data sets such as our recently published CompaCt tool,^38^ or methods that integrate additional sources of interaction evidence such as EPIC^8^ and ComplexFinder,^7^ are able to robustly identify complexes that are consistently detected across multiple complexome profiling experiments or for which we have other types of interaction evidence. In contrast, the GIP is useful for the identification of complexes that are not consistently identifiable across a large collection of studies. These could include complexes that are hard to detect with standard complexome profiling protocols, such as lowly abundant complexes or strongly hydrophobic interactors. Similarly, the GIP can enable identification of complexes that are present in a specific system under certain conditions that have not yet been extensively studied, or complexes that are uniquely detected using new experimental protocols.

The GIP is the only tool that provides means of prioritizing clusters, and we show that high-scoring clusters are more likely to present actual protein complexes. Manual inspection of often hundreds of clusters to identify potentially novel complexes or interactors is difficult, time-consuming and inherently biased, especially in the case of smaller complexes. The prioritization of clusters and complex members enables straightforward identification of the most pronounced results, which will help with discovery of novel candidate protein complexes and interactions from complexome profiles.

In some cases, the protein subunits that make up a protein complex have differences in their migration patterns in complexome profiling. This is usually caused by the presence of subassemblies of the protein complex, which are represented by additional peaks in the migration pattern of a subset of the complex subunits. Due to these differences some protein complexes tend to be split across multiple clusters. For example, in one of the analyzed human complexome profiles, the 20S core of the proteasome is in one cluster, while the regulatory subunits are in another one (Supporting Information Figure S6A). Similarly, the subunits of the mitochondrial ribosome have been split across two clusters, representing the large and small ribosomal subunits (Supporting Information Figure S6B). While this phenomenon can complicate the identification of complete complexes, it does indicate differences in the migration of its subunits, which can point toward their presence in a different interaction and provide insight in complex assembly.

Finally, we demonstrate that the GIP can be a valuable tool to explore the interplay of proteins in the malaria parasite, suggesting associations that could provide novel insights into the parasite’s divergent biology and highlighting potential new targets for antimalarial interventions that would have been easily overlooked with conventional analysis approaches. While this study provides several promising starting points, it is important to keep in mind that the interactions identified by the GIP are putative and require further validation. Nonetheless, these findings provide a valuable resource for the malaria research community, specific leads for wet-lab investigation and illustrate the power of complexome profiling combined with GIP-based analysis, not only in shedding light on the malaria parasite’s complex biology, but also to facilitate the reliable identification of novel protein–protein interactions.

## Supporting Information Available

Figure S1: Benchmarking cut point for hierarchical clustering of complexome profiles; Figure S2: Benchmarking ClusterOne parameters for clustering complexome profiles; Figure S3: per-sample performance of the tested clustering methods; Figure S4: The number of identified clusters for each tested clustering method; Figure S5: The relationship between the abundance of protein complexes and their recovery by the GIP; Figure S6: Visualization of the migration patterns of four GIP result clusters clusters; Table S1: Pairwise (single-sample) *t* test, difference in mmr between clustering methods (PDF)
Details of clusters and clustered proteins resulting from GIP analysis of Plasmodium complexome profiles (XLSX)
Membrane Mass calibration of *Plasmodium* complexome profiles (XLSX)
