# Supplementary material for: Analysis of Complexome Profiles with the Gaussian Interaction Profiler (GIP) Reveals Novel Protein Complexes in Plasmodium falciparum
Source: J Proteome Res. 2024 Sep 12;23(10):4467–79. doi: 10.1021/acs.jproteome.4c00414 (PMC11459595; doi:10.1021/acs.jproteome.4c00414)
Supplement: Supplementary file 1 — pr4c00414_si_001.pdf [file pr4c00414_si_001.pdf]

Supporting information for: Analysis of complexome profiles with the Gaussian Interaction Profiler (GIP) reveals novel protein complexes in *Plasmodium falciparum*

Joeri van Strien [1#], Felix Evers[2#], Alfredo Cabrera-Orefice[1], Iris Delhez[1], Taco WA Kooij[2#], Martijn A. Huynen[1*#]

[1] Department of Medical BioSciences, Radboud University Medical Center, 6500 HB Nijmegen, the Netherlands
[2] Medical Microbiology, Radboud Community for Infectious Diseases, Radboud University Medical Center, 6500 HB Nijmegen, The Netherlands

*corresponding author.
[#]these authors contributed equally to this work
email: Martijn.Huijnen@radboudumc.nl

Table of contents

Figure S1. Benchmarking cutpoint for hierarchical clustering of complexome profiles

Figure S2. Benchmarking ClusterOne parameters for clustering complexome profiles

Figure S3. per-sample performance of the tested clustering methods

Figure S4. The number of identified clusters for each tested clustering method

Figure S5. The relationship between the abundance of protein complexes and their recovery by GIP

Figure S6. Visualization of the migration patterns of four GIP result clusters clusters.

Table S1. Pairwise (single-sample) t-test, difference in mmr between clustering methods

Table S2. Details of clusters and clustered proteins resulting from GIP analysis of *Plasmodium* complexome profiles. (xlsx)

Supplementary File S2. Membrane Mass calibration of *Plasmodium* complexome profiles. (xlsx)

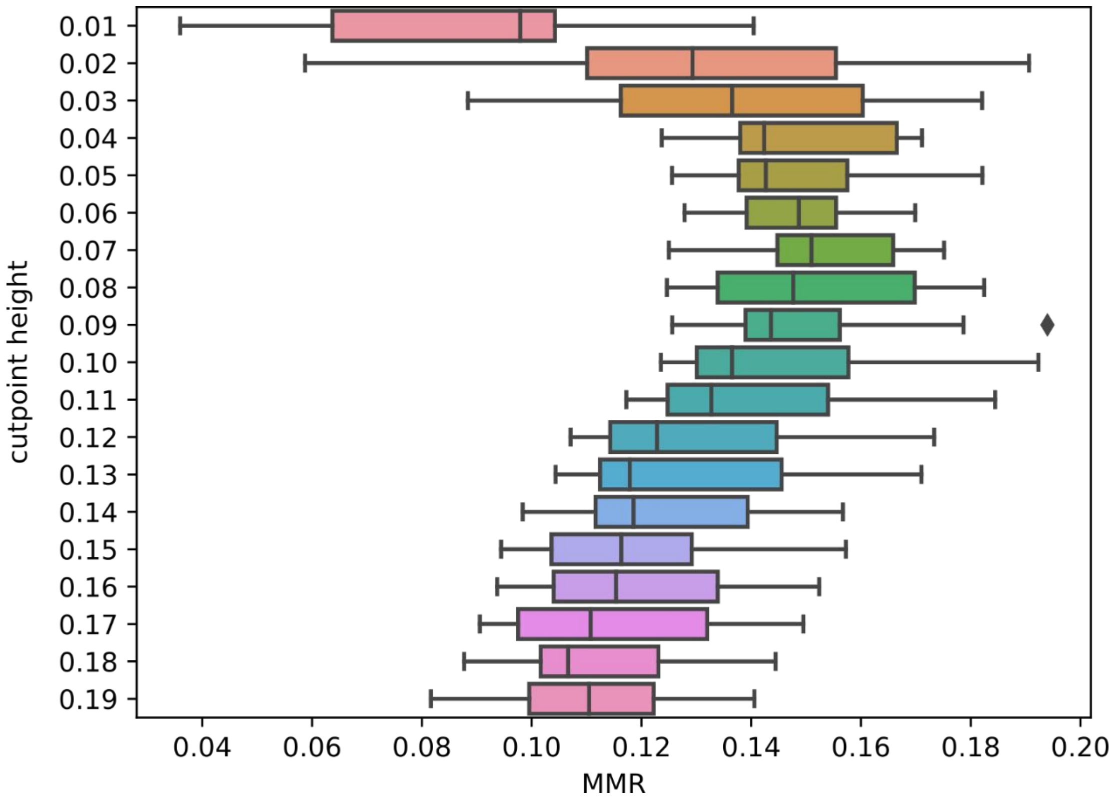

Figure S1: Benchmarking the optimal cutpoint for hierarchical clustering of complexome profiles. Average linking hierarchical clustering [1] was performed on eight human fibroblast complexome profiles, using Pearson correlation as a similarity measure between protein migration patterns. A range of cutpoints was taken to generate discrete clusters. For each cutpoint and complexome profiling sample the agreement of the cluster results with a set of CORUM [2] reference complexes was determined using the maximum matching ratio [3]. The cutpoint at a height of 0.07 resulted in the highest average maximum matching ratio (MMR) over all eight clustered complexome profiles.

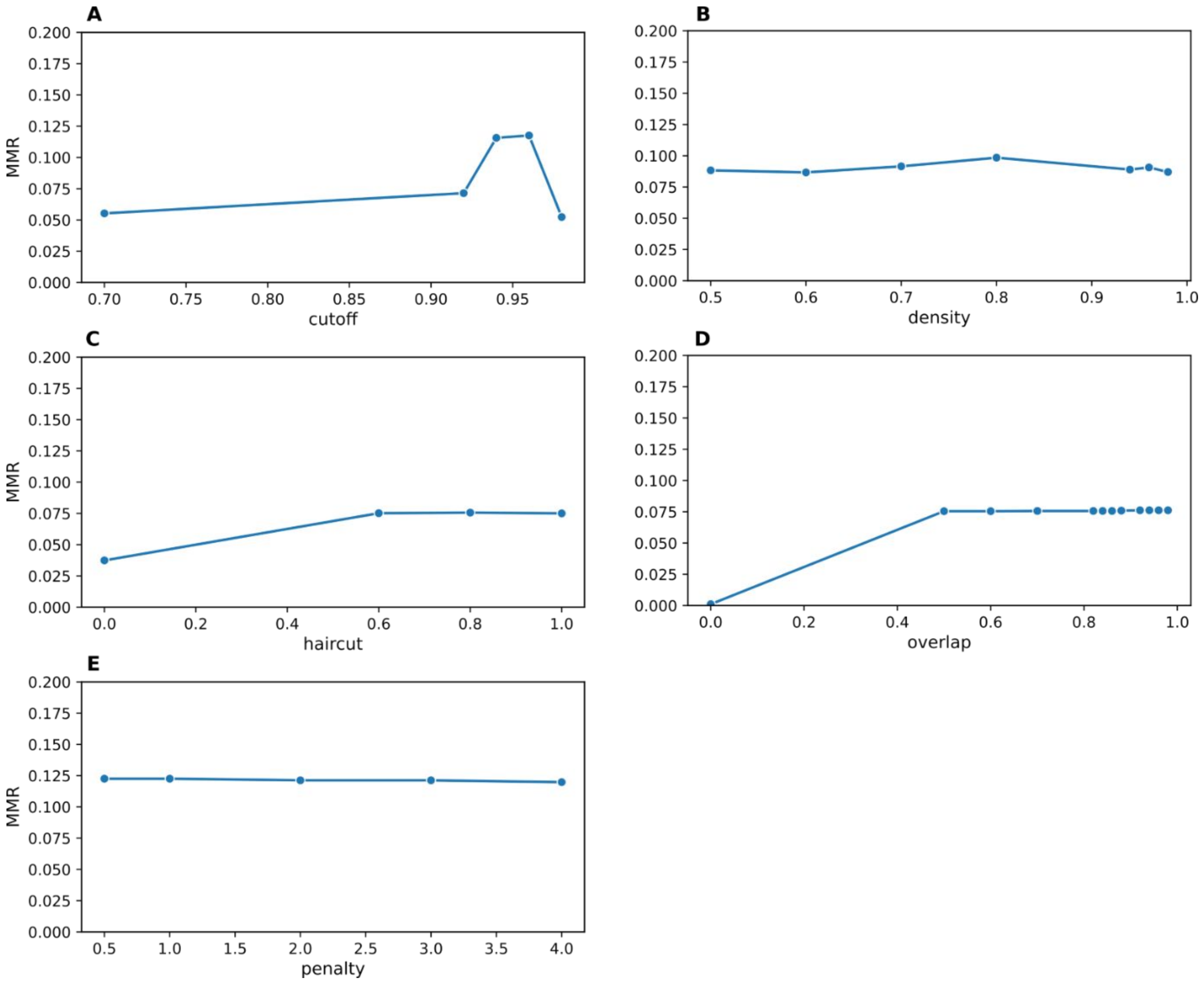

Figure S2: Benchmarking of optimal ClusterOne parameters for clustering complexome profiles. ClusterOne was applied to the CRS17 human fibroblast complexome profile [4]. The recovery of a set of CORUM [2] complexes with ClusterOne was determined using the maximum matching ratio for a range of parameter values [3]. A) Performance of a range of Pearson correlation threshold values for inclusion of edges in the ClusterOne input network. B) Performance of a range of values of the “density” parameter. C) Performance of a range of values of the “haircut” parameter. D) Performance of a range of values of the “overlap” parameter. E) Performance of a range of values of the “penalty” parameter. Abbreviations: MMR: maximum matching ratio.

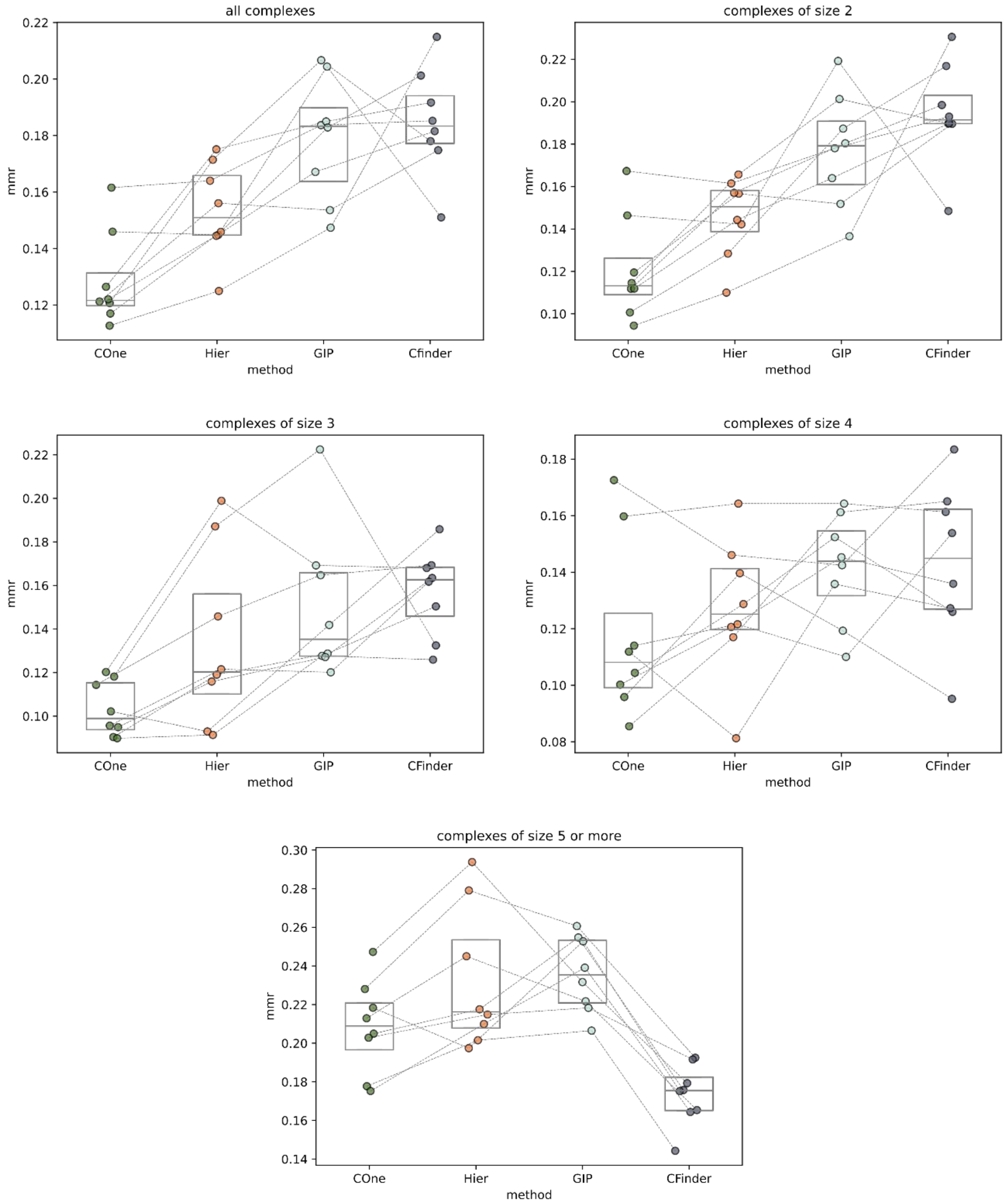

Figure S3: Sample-linked performance of of each tested clustering method in recovering known protein complexes, expressed in the maximum matching ratio (mmr, y-axis) of the clusters compared to a detected set of CORUM protein complexes. The boxes represent the median and quartiles. Each dot represents a single complexome profiling sample. Connected dots represent the same complexome profiling sample clustered by different methods. The panels display the performance for either all tested protein complexes, or a subset of the protein complexes falling within a specific size category.

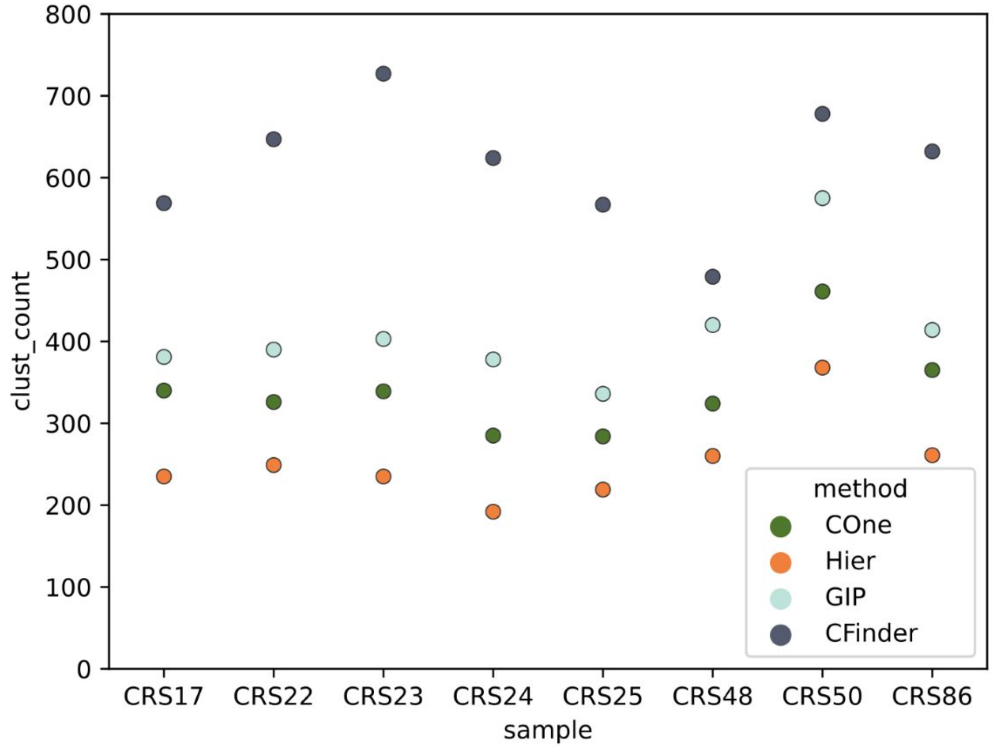

Figure S4: The number of identified clusters for each tested clustering approach (ClusterOne, hierarchical clustering, GIP clustering and ComplexFinder). The numbers shown are the total number of identified of clusters containing at least two proteins identified by each clustering approach on each human complexome profile.

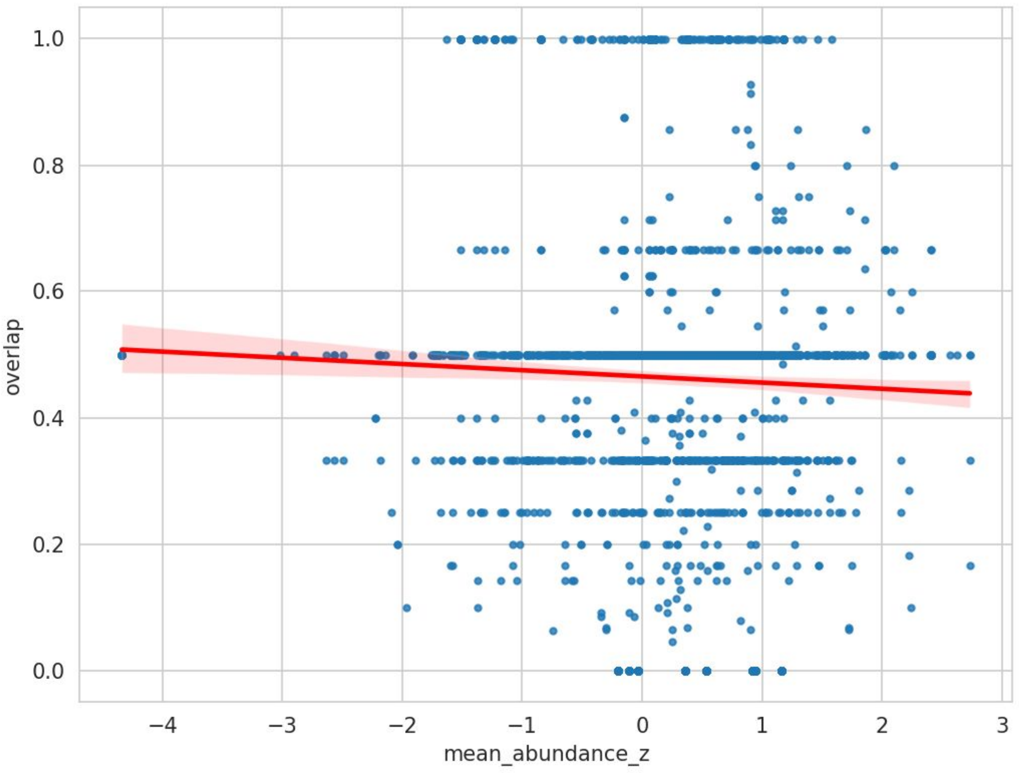

Figure S5: Scatterplot showing the relationship between the mean abundance of known protein complexes and their recovery by a GIP result cluster. Dots represent the set of GIP output clusters that show the highest overlap with one of the CORUM reference complexes within each complexome profile, for eight analysed human fibroblast complexome profiles. The x-axis shows the mean log-normalized z-scored abundance of each detected protein in the respective complexome profile. The y-axis shows the overlap between the GIP cluster and the corresponding reference complex. The red line shows the linear relationship between the mean abundance and the overlap, with a 95% confidence interval shown as semi-transparent red.

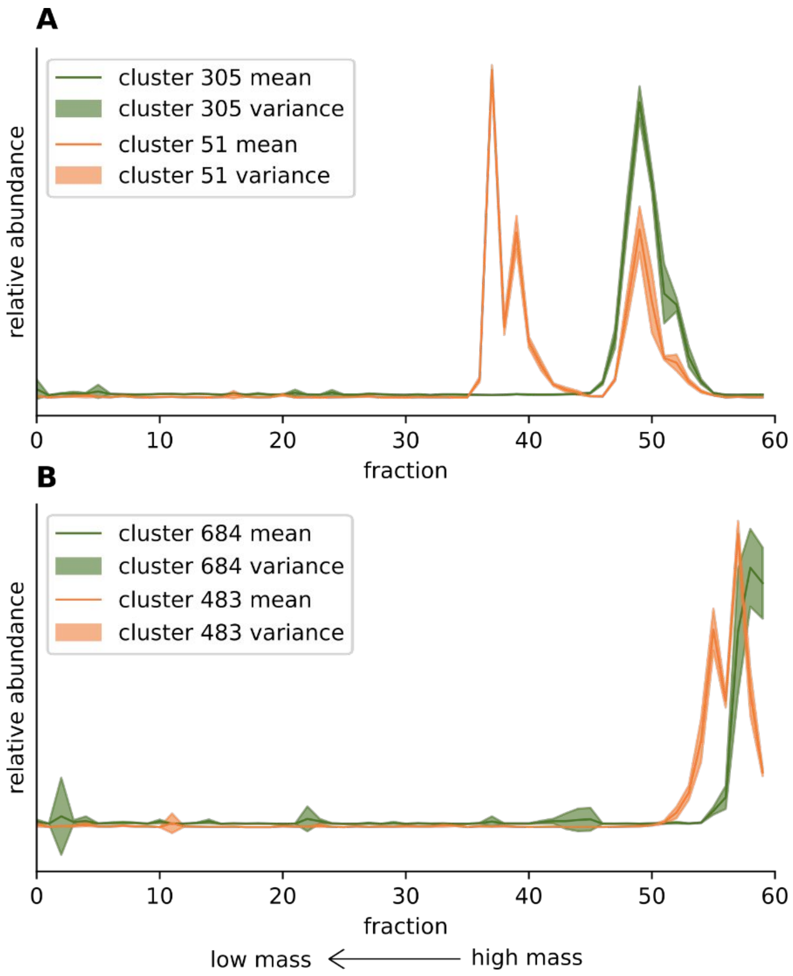

Figure S6: Vizualization of the migration patterns of four GIP result clusters clusters. The mean +- the variance for each fraction from the fit Gaussian mixture model are shown for each cluster. A) Two clusters containing subunits from different components of the proteasome. Cluster 51 contains subunits from the 20S core component of the proteasome, while cluster 305 contains regulatory subunits. B) Two clusters containing subunits from the mitochondrial ribosome. Cluster 684 contains subunits from the large 60S ribosomal subunit, while cluster 483 contains subunits from the small 40S ribosomal subunit.

Table S1. Pairwise (single-sample) t-test, of the difference in maximum matching ratio (mmr), a measure for the recovery of known protein complexes by the various tested clustering methods. P-values below 0.05 are highlighted in green.

**complex size category**	**difference in mmr**	**p-value (difference > 0)**
all complexes	GIP - COne	**<0,001**
	GIP - Hier	**0,003**
	CFinder - GIP	0,326
size 2	GIP - COne	**<0,001**
	GIP - Hier	**0,005**
	CFinder - GIP	0,165
size 3	GIP - COne	**<0,001**
	GIP - Hier	0,054
	CFinder - GIP	0,334
size 4	GIP - COne	**0,044**
	GIP - Hier	0,093
	CFinder - GIP	0,414
size 5+	GIP - COne	**0,008**
	GIP - Hier	0,406
	GIP - CFinder	<0,001
